# Healthy-lifestyle behaviors associated with overweight and obesity in US rural children

**DOI:** 10.1186/1471-2431-12-102

**Published:** 2012-07-18

**Authors:** Alison Tovar, Kenneth Chui, Raymond R Hyatt, Julia Kuder, Vivica I Kraak, Silvina F Choumenkovitch, Alia Hastings, Julia Bloom, Christina D Economos

**Affiliations:** 1 John Hancock Research Center on Physical Activity, Nutrition and Obesity Prevention, Gerald J. and Dorothy R. Friedman School of Nutrition Science and Policy, Tufts University, 150 Harrison Ave., Boston, MA 0211, USA; 2 Department of Public Health and Community Medicine, Tufts University, 136 Harrison Avenue, Boston, MA 02111, USA; 3 Deakin Population Health Strategic Research Centre, School of Health and Social Development, Bldg E 1.08, Faculty of Health, Deakin University, 221 Burwood Highway, Burwood, Victoria, 3125, Australia

**Keywords:** Obesity, Children, Rural, Diet, Physical activity, Vulnerable populations, Healthy lifestyle behaviors

## Abstract

**Background:**

There are disproportionately higher rates of overweight and obesity in poor rural communities but studies exploring children’s health-related behaviors that may assist in designing effective interventions are limited. We examined the association between overweight and obesity prevalence of 401 ethnically/racially diverse, rural school-aged children and healthy-lifestyle behaviors: improving diet quality, obtaining adequate sleep, limiting screen-time viewing, and consulting a physician about a child’s weight.

**Methods:**

A cross-sectional analysis was conducted on a sample of school-aged children (6–11 years) in rural regions of California, Kentucky, Mississippi, and South Carolina participating in CHANGE (Creating Healthy, Active, and Nurturing Growing-up Environments) Program, created by Save the Children, an independent organization that works with communities to improve overall child health, with the objective to reduce unhealthy weight gain in these school-aged children (grades 1–6) in rural America. After measuring children’s height and weight, we17 assessed overweight and obesity (BMI ≥ 85th percentile) associations with these behaviors: improving diet quality18 (≥ 2 servings of fruits and vegetables/day), reducing whole milk, sweetened beverage consumption/day; obtaining19 adequate night-time sleep on weekdays (≥ 10 hours/night); limiting screen-time (i.e., television, video, computer,20 videogame) viewing on weekdays (≤ 2 hours/day); and consulting a physician about weight. Analyses were adjusted 21 for state of residence, children's race/ethnicity, gender, age, and government assistance.

**Results:**

Overweight or obesity prevalence was 37 percent in Mississippi and nearly 60 percent in Kentucky. Adjusting for covariates, obese children were twice as likely to eat ≥ 2 servings of vegetables per day (OR=2.0,95% CI 1.1-3.4), less likely to consume whole milk (OR=0.4,95% CI 0.2-0.70), Their parents are more likely to be told by their doctor that their child was obese (OR=108.0,95% CI 21.9-541.6), and less likely to report talking to their child about fruits and vegetables a lot/sometimes vs. not very much/never (OR=0.4, 95%CI 0.2-0.98) compared to the parents of healthy-weight children.

**Conclusions:**

Rural children are not meeting recommendations to improve diet, reduce screen time and obtain adequate sleep. Although we expected obese children to be more likely to engage in unhealthy behaviors, we found the opposite to be true. It is possible that these groups of respondent parents were highly aware of their weight status and have been advised to change their children’s health behaviors. Perhaps given the opportunity to participate in an intervention study in combination with a physician recommendation could have resulted in actual behavior change.

## Background

While the overweight and obesity crisis currently affects nearly one third of American children and adolescents, ages 2 to 19 years [1], the prevalence is considerably higher for ethnically and racially diverse children and adolescents [1,2] and low-income children in rural regions of the United States [3-8]. The disproportionately higher rates of overweight and obesity in poor rural communities are attributed to several environmental factors: limited availability of and access to healthy foods and beverages, fewer physical activity opportunities; limited access to health care services, low population density that prohibits safe walking, “food deserts” that require residents to travel long distances to purchase affordable healthy foods, and a lack of recreation centers [6,9-11].

Two recent studies have documented heightened concern about overweight and obesity among parents, children and teachers in rural Appalachia (term used to describe a cultural region in the eastern United States that stretches from the Southern Tier of New York state to northern Alabama, Mississippi, and Georgia), where children’s and parents’ obesity rates exceed national estimates [12,13]. Despite the ecological and environmental relationships that have been documented in the literature, studies exploring children’s health-related behaviors that may assist in designing effective interventions for poor rural populations are limited.

The goal of this paper was to examine the association between overweight and obesity prevalence of 401 ethnically and racially diverse, rural school-aged children and their healthy-lifestyle behaviors. The children were enrolled in a two-year, randomized controlled trial called the Creating Healthy, Active and Nurturing Growing-up Environments (CHANGE) Study implemented in rural regions of four states: California, Kentucky, Mississippi and South Carolina. The behaviors of interest include: improving diet quality, obtaining adequate sleep, limiting screen-time viewing, and parents consulting a physician about their child’s weight status. We hypothesized that overweight and obese children would be more likely to engage in unhealthy lifestyle behaviors, compared to normal weight children.

## Methods

In 2005, Save the Children (a leading independent organization that works with communities, to help children and families; they work with other organizations, governments, non-profits and a variety of local partners while maintaining their own independence without political agenda or religious orientation) created the CHANGE (Creating Healthy, Active, and Nurturing Growing-up Environments) Program whose objective was to reduce unhealthy weight gain in school-aged children (grades 1–6, ages 6–11 years) in rural America. Children participating in the CHANGE program engaged in 30 minutes or more of moderate –to-vigorous physical activity per day and ate healthy snacks to improve dietary intake. To evaluate their efforts, a partnership between Save the Children and the Friedman School of Nutrition Science and Policy at Tufts University was formed and a randomized controlled, community-based participatory research study was conducted. The goal of the CHANGE Study, conducted from 2007 to 2009, was to adapt, replicate, and evaluate the *Shape Up Somerville* childhood obesity intervention [14] in four rural regions of California (Central Valley), Kentucky (Appalachia), Mississippi (Delta), and South Carolina [15]. These regions were selected by Save the Children for their high poverty rate and their connection with Save the Children regional offices and although from rural areas they do not represent all of the rural regions in the US. The Institutional Review Board at Tufts University approved the recruitment and study procedures. Informed written consent was obtained from all participating children’s parents or guardians and child assent was obtained for children over the age of 7 years.

For this study, we used data collected at baseline from 8 randomly selected elementary schools from rural communities in South Carolina (SC), Mississippi (MS), Kentucky (KY), and California (CA). A total of 1235 children were recruited. We included baseline data collected in 2008 (67% collected between January and May 2008 and 33% collected between September and December 2008). Case exclusion was performed based on the following criteria: parents/guardians did not return the family survey (788 excluded), missing children’s age (35), missing children’s height and weight data (4), and BMI z-score less than 5^th^ percentile, i.e. underweight (9). We omitted the underweight children given their very low proportion (approximately 2% of the final samples). This resulted in a sample size of 401, the final sample for this analysis.

### Measures

Child demographic information was collected at the time of informed consent. Demographic information collected included race/ethnicity (i.e., White, Black, Hispanic, Asian, multiracial, or other), age, child’s grade, and gender. Baseline measures for children’s height and weight were obtained in triplicate, without shoes, by trained staff following recommended procedures for standardized anthropometric measurement in school settings, as previously described [14]. Height was measured to the nearest eighth of an inch using a portable stadiometer (Shorr Height/Length Measuring Board; Shorr, Olney, MD) and weight was measured in light clothing to the nearest 0.5 lb on a digital scale (Seca Bella model 480; Seca, Hanover, MD). Body Mass Index (BMI) was calculated using an average of three body weight and height measures within the ranges cited above, and converted to z-scores as previously described [14]. In accordance with CDC guidelines, children with a z-score below the 5^th^ percentile were considered underweight, those with a z-score between the 5^th^ and 85^th^ percentiles were considered healthy weight, those with a z-score at or above the 85^th^ percentile but below the 95^th^ percentile were considered overweight, and those with a z-score at or above the 95^th^ percentile were considered obese [16].

A 41-item Family Survey Questionnaire was constructed using validated questions from different surveys and was mailed to parents or guardians (Table 1) and had a 36 percent response rate.

**Table 1:** Sections from the 41-item Family Survey Questionnaire

**Sections**	**Types of Questions**
1) Child’s eating and drinking habits	Questions about fruit, vegetable, dairy, soda, snack-food consumption and family meals; parents reported the number of servings of fruits and vegetables eaten by their children in a typical day (0 to 5 or more).
	Questions on the number of 12-ounce servings of sweetened soda and other sugar-sweetened beverages (i.e., Kool-aid, sport drinks) their child drank per day or per week.
	Beverage questions were adapted from the Harvard Service Food Frequency Questionnaire [17]
2) Child’s non-active time habits	Questions on sleep, screen-time (i.e., television, video, computer, and video game) exposure, reported as total hours and/or minutes per week), number of televisions in the household and eating in front of the TV during meals using questions in a standard, validated format[18]
3) Child’s physical activity habits and parental support questions	Reporting of organized sports and physical activity. Does your child participate in organized sports (Yes/No). What sports reported by season.
	How often do you encourage your child to be physically active (a lot/sometimes/rarely/never)
	How often do you and your child do something active together (a lot/sometimes/rarely/never)
	How often do you and your child talk about fruits and vegetables (a lot/sometimes/rarely/never)
4) Child’s medical information	“Has your child’s doctor ever told you that your child is overweight or obese?”
5) Household information	Parental education (i.e., less than high school, high school, some college, or college/graduate school) for parents, parental marital status, number of children in household, parental place of birth, and whether the family received government assistance.
	Parental education and government assistance were used as proxy measures for socio-economic status given that collecting verifiable information about household income was not feasible in this rural sample.

### Statistical analyses

We conducted all analyses using SAS version 9.0 (SAS Institute, Cary, NC). We first examined child and parental characteristics according to weight status (healthy weight vs. overweight/obese). We used one-way analysis of variance to compare means for continuous variables of different groups, and χ^2^ test to examine differences in categorical variables. We then compared how children in each weight category complied with various recommendations for health-related behaviors. To further understand the association between reported practicing of certain health behaviors and a set of selected predictors based on our conceptual framework, we performed a series of logistic regression analyses. Behaviors that were significantly (p<0.10) predicted by weight status in the previous analysis were included as categorical outcome variables. We selected the following outcome variables: vegetable and whole milk consumption, hours of sleep, “has your doctor ever told you that your child is overweight or obese”, “how often do you and your child do something active together”, and “how often do you and your child talk about fruits and vegetables”. Using SAS proc LOGISTIC , we fit logistic regression models with overweight and obese being the main predictor variables (Overweight/Obese = 1, Normal weight = 0. Underweight excluded). Confounding factors (selected based on previous literature) in the final models were age, gender, race/ethnicity, state of residence, number of members in the household and whether the family received government assistance. Since number of members in the household had a larger number of missing values, a missing value category was created and used to retain the sample size for analysis.

## Results

Overall, out of a total of 401 children in 1^st^ through 6^th^ grade, 49.5% were female; 46.8% were Black; 34.7% were Hispanic; 20% of mothers reported having less than a high school degree and almost 50% reported receiving government assistance (Table 2). Approximately 30% of mothers were not born in the US and 27.2% reported that Spanish was the primary language spoken at home. Forty-five percent of children were overweight and/or obese (defined as BMI z-score above the 85^th^ percentile for age and sex) and 23% had a BMI z-score above the 97^th^ percentile for age and sex. Of the 45 participants enrolled from Kentucky almost 60% were overweight or obese, in California, of 152 children, 50% of children were overweight or obese, in South Carolina of the 73 enrolled, 44% were overweight or obese and finally of the 131 enrolled in Mississippi, 37% were overweight or obese. Having more than 4 members in the household (49.7% for healthy weight versus 43.2% and 30.9% for overweight and obese, respectively) differed significantly across weight categories.

**Table 2:** Sample Description of Participants’ Characteristics according to Weight Status n,%

		**All participants**		**Healthy weight**		**Overweight**		**Obese**	
**Gender**									
	**Male**	203	50.5	108	49.5	35	53.0	59	51.3
	**Female**	198	49.5	110	50.5	31	47.0	56	48.7
**Age in years, mean (SD)**		9.3	1.7	9.2	1.8	9.2	1.6	9.4	1.7
**State**									
	**California**	152	37.9	77	35.3	30	45.5	46	40.0
	**Kentucky**	45	11.1	20	9.2	9	13.6	16	13.9
	**Mississippi**	131	32.7	81	37.2	17	25.8	31	27.0
	**South Carolina**	73	18.0	40	18.3	10	15.5	22	19.1
**Reported race/ethnicity**									
	**White**	56	13.8	30	13.7	9	13.6	17	14.8
	**Black**	190	46.8	111	50.9	23	34.9	52	45.2
	**Hispanic**	141	34.7	68	31.2	28	42.4	42	36.5
	**Other**	19	4.7	9	4.1	6	9.0	4	3.5
**Maternal education level**									
	**Less than high school**	66	20.0	30	17.4	13	25.0	22	22.0
	**High school graduate**	108	32.7	52	30.2	14	26.9	41	41.0
	**Technical school/Associates degree**	90	27.3	53	30.8	14	26.9	20	20.0
	**College graduate**	42	12.7	22	12.8	8	15.4	11	11.0
	**Graduate**	24	7.3	15	8.7	3	5.8	6	6.0
**Mother born in the US**									
	**No**	122	30.4	59	27.2	21	32.8	39	34.5
	**Yes**	279	69.6	158	72.8	43	67.2	74	65.5
**English as primary language**									
	**English**	284	72.8	164	77.0	43	67.2	73	67.6
**Spanish as primary language**									
	**Spanish**	106	27.2	49	23.0	21	32.8	35	32.4
**Receiving government assistance**									
	**No**	203	51.3	108	50.5	36	56.3	55	49.5
	**Yes**	193	48.7	106	49.5	28	43.8	56	50.5
**Marital status**									
	**Never married**	99	24.9	62	28.8	12	18.8	22	19.8
	**Married**	244	61.5	129	60.0	44	68.8	68	61.3
	**Separated/Divorced**	43	10.8	21	9.8	5	7.8	16	14.4
	**Widowed**	11	2.8	3	1.4	3	4.7	5	4.5
Number of household members*									
	**4 or less**	172	56.6	88	50.3	25	56.8	56	69.1
	**More than 4**	132	43.7	87	49.7	19	43.2	25	30.9

*p<0.05, based on Chi-square test.

Almost two thirds (64.7%) of parents reported that their child watched more than two hours of screen time per weekday (Table 3) and almost three quarters of parents surveyed reported that their child sleeps less than the recommended 10 hours per night, the average hours of sleep for our sample was 9.4. Parental surveys revealed that approximately 40% of children eat less than two servings of fruits and vegetables per day and only 15% of children drink 1% low-fat milk and 2.8% drinks nonfat milk. Almost all of the parents (96.2%) reported that their child drinks at least one can of soda per day and one can of a sugar-sweetened beverage (not including soda, i.e Hi-C, Kool-aid, sports drinks).

**Table 3:** Comparison of Compliances to Healthy Behavior Recommendations between Children in Healthy, Overweight and Obese Categories

**n,(%)**	**All children**	**Healthy weight**	**Overweight**	**Obese**
Hours of sleep per night*				
**<10 hrs/night**	269 (69.7)	162 (76.1)	31 (48.4)	76(69.7)
**≥10 hrs/night**	117 (30.3)	51 (23.9)	33 (51.6)	33(30.3)
**Hours of screen-time on weekday**				
**≤2 hrs/day**	117 (35.3)	57 (32.4)	25 (41.7)	35 (36.8)
**>2 hrs/day**	214 (64.7)	119 (67.6)	35 (58.3)	60 (63.2)
**Servings of fruit per day**				
**<2**	142 (36.3)	83 (38.8)	21(32.8)	37 (33.3)
**≥2**	249 (63.7)	131 (61.2)	43(67.2)	74 (66.7)
**Servings of vegetables per day**				
**<2**	153 (39.3)	95 (44.4)	21 (33.3)	39 (33.6)
**≥2**	236 (60.7)	119 (55.6)	43 (66.7)	77 (66.4)
My child drinks whole milk*				
**No**	245 (62.3)	118 (54.9)	42 (64.6)	85 (75.2)
**Yes**	148 (37.7)	97 (45.1)	23 (35.4)	28 (24.8)
My child drinks low fat milk 1%*				
**No**	335 (85.2)	187 (86.9)	60 (92.3)	88 (77.8)
**Yes**	58 (14.7)	28 (13.1)	5 (7.7)	25 (21.0)
**My child drinks non-fat milk**				
**No**	382 (97.2)	212 (98.6)	64 (98.5)	106 (93.8)
**Yes**	11 (2.8)	3 (1.4)	1 (1.5)	7 (6.2)
**Number of Soda cans per day**				
**<1 can/day**	5 (3.8)	3 (4.5)	0	2 (5.1)
**≥1 can/day**	127 (96.2)	64 (95.5)	26 (100.0)	37 (94.9)
**Number of sugar-sweetened drink cans per day**				
**<1 can/day**	3 (2.0)	1 (1.2)	0	2 (4.2)
**≥1 can/day**	149 (98.0)	82 (98.8)	21 (100.0)	46 (95.8)

*p<0.05 based on Chi-square test.

**Table 4:** Parental Reported Health Behaviors of children in the Healthy, Overweight and Obese Categories

**n,%**	**All children**	**Healthy weight**	**Overweight**	**Obese**
**On a typical school day does your child eat breakfast**				
**Yes at home**	124 (34.4)	69 (35.0)	24 (41.4)	31 (29.3)
**Yes, at school**	229 (63.4)	125 (63.5)	32 (55.2)	72 (67.9)
**No, my child does not eat breakfast**	8 (2.2)	3 (1.5)	2 (3.5)	3 (2.8)
**How many times in the past week family meal together**				
**≤3 times/week**	186 (47.5)	106 (48.9)	33 (52.4)	47 (42.0)
**>3 times/week**	206 (52.6)	111 (51.2)	30 (47.6)	65 (58.0)
**Do you have a TV in your bedroom?**				
**Yes**	325 (81.5)	186 (85.3)	50 (75.7)	89 (77.4)
**No**	74 (18.6)	32 (14.7)	16 (24.2)	26 (22.6)
**Is there a TV where your child sleeps?**				
**Yes**	270 (68.8)	155 (71.4)	37 (57.8)	78 (70.3)
**No**	122 (31.1)	62 (28.6)	27 (42.2)	33 (29.7)
**Number of TV's in the household**				
**1**	74 (18.7)	38 (17.5)	16 (24.2)	20 (17.9)
**2**	226 (57.2)	130 (59.9)	32 (48.5)	64 (57.1)
**≥3**	95 (24.1)	49 (22.6)	18 (27.3)	28 (25.0)
**Child Participate in After School Program?**				
**Yes**	112 (29.2)	58 (27.1)	21 (33.3)	33 (30.8)
**No**	272 (70.8)	156 (72.9)	42 (63.7)	74 (69.2)
**It is safe for my child to play outdoors w/out adult**				
**Strongly disagree/Somewhat Disagree**	244 (63.5)	136 (64.2)	38 (60.3)	70 (64.2)
**Neutral**	40 (10.4)	26 (12.3)	6 (9.5)	8 (7.3)
**Somewhat agree/Strongly Agree**	100 (26.0)	50 (23.6)	19 (30.2)	31 (28.4)
**How often do you encourage your child to be physically active**				
**A lot**	247 (62.7)	141 (65.9)	36 (54.6)	70 (61.4)
**Sometimes**	126 (32.0)	61 (28.5)	25 (37.9)	40 (35.1)
**Rarely**	15 (3.8)	9 (4.2)	2 (3.0)	4 (3.5)
**Never**	6 (1.5)	3 (1.4)	3 (4.6)	0
How often do you and your child do something active together?*				
**A lot**	130 (32.8)	79 (36.7)	16 (24.2)	35 (30.4)
**Sometimes**	222 (56.1)	104 (48.4)	45 (68.2)	73 (63.5)
**Rarely**	37 (9.3)	26 (12.1)	5 (7.6)	6 (5.2)
**Never**	7 (1.8)	6 (2.8)	0	1 (0.8)
How often do you and your child talk about fruits and vegetables?*				
**A lot**	151 (38.2)	81 (37.7)	19 (28.8)	51 (44.7)
**Sometimes**	185 (46.8)	96 (44.7)	36 (54.6)	53 (46.5)
**Rarely**	42 (10.6)	23 (10.7)	10 (15.2)	9 (7.9)
**Never**	17 (4.3)	15 (7.0)	1 (1.5)	1 (0.9)
Has your child's doctor ever told you that your child is overweight or obese?*				
**No**	341 (87.0)	212 (99.1)	59 (90.8)	70 (61.9)
**Yes**	51 (13.0)	2 (0.9)	6 (9.2)	43 (38.1)

*p<0.05, based on Chi-square test.

**Table 5:** Odds Ratios of Selected Health Behaviors According to Overweight and Obesity Status Compared to Healthy Weight

**Predictor**	**Being Overweight Unadjusted**			Being Overweight Adjusted*			**Being Obese Unadjusted**			Being Obese Adjusted*		
	**OR**	**95%CI**	**P-value**	**OR**	**95%CI**	**P-value**	**OR**	**95%CI**	**P-value**	**OR**	**95%CI**	**P-value**
**Vegetable Consumption**												
**< 2servings/day**	Ref.	-	-	Ref.	-	-	Ref.	-		Ref.	-	
**≥2 servings/day**	1.6	0.9-3.0	0.10	1.6	0.8-2.9	0.2	1.6	0.98- 2.5	0.06	2.0	1.1-3.4	0.02
**Whole Milk**												
**No**	Ref.	-	-	Ref.	-	-	Ref.	-	-	Ref.	-	-
**Yes**	0.7	0.4-1.2	0.2	0.9	0.4-1.8	0.8	0.4	0.2-0.7	<0.001	0.4	0.2-0.7	0.001
**Sleep**												
**<10hrs/night**	Ref.	-	-	Ref.	-	-	Ref.	-	-	Ref.	-	-
**≥10 hrs/night**	3.4	1.9-6.0	<0.001	3.7	1.9-7.2	<0.001	1.3	0.8-2.3	0.3	1.3	0.7-2.4	0.3
**Ever told by doctor that your child was Overweight/obese?**												
**No**	Ref.	-	-	Ref.	-	-	Ref.	-	-	Ref.	-	-
**Yes**	10.8	2.1- 54.5	0.004	10.2	1.7-59.2	0.009	64.8	15.3-274.0	<0.0001	108.0	21.9-541.6	<0.0001
**How often do you and your child talk about fruits and vegetables?**												
**A lot/Sometimes**	0.9	0.4- 1.9	0.4	0.9	0.4-2.1	0.8	0.4	0.2- 0.9	0.04	0.4	0.2-0.98	0.04
**Not very much/Never**	Ref.	-		Ref.	-	-	Ref.	-	-	Ref.	-	-

*All models adjusted for state, race/ethnicity, gender, age, number of members in household and government assistance.

With regard to children’s eating behaviors reported by parents, 63% reported that their child eats breakfast at school and almost 50% reported that they eat family meals together less than three times per week (Table 4).Eighty-two percent of parents reported that they have a TV in their bedroom and nearly 68.8% reported that their child has a TV in his/her own bedroom. Almost 60% of parents reported having two TVs in their home and one quarter reported having more than 3 TVs in their home. Seventy-one percent of children did not participate in afterschool programs and 63.5% of parents strongly disagreed with the statement “it’s safe for my child to play outside.”

In unadjusted analysis (shown in Table 5), we found that children who are obese compared to those who are within a healthy weight range were more likely to eat **≥** 2 servings of vegetables per day (OR=1.6,95% CI 0.98-2.5), less likely to drink whole milk (OR=0.4,95% CI 0.2-0.7), more likely to be told by their doctor that their child was obese (OR=65.1,95% CI 15.3-274.0) and less likely for parents to report talking to their child about fruits and vegetables a lot/sometimes vs. not very much/never (OR=0.4, 95%CI 0.2-0.9). Children who are overweight compared to those who are within a healthy weight range were more likely to sleep ≥10hrs/night (OR=3.4,95%CI 1.9-6.0). Adjusting for state of residence, age, gender, number of members in the household, and government assistance, obese children were still more likely to eat ≥ 2 servings of vegetables per day (OR=2.0,95% CI 1.1-3.4), less likely to consume whole milk (OR=0.4,95% CI 0.2-0.7), more likely to be told by their doctor that their child was obese (OR=108.0,95% CI 21.9-541.6), and less likely for their parents to report talking to their child about fruits and vegetables a lot/sometimes vs. not very much/never (OR=0.4, 95%CI 0.2-0.98).

## Discussion

In this cross-sectional baseline analysis we examined healthy-lifestyle behaviors of overweight and obese children compared to healthy weight children enrolled in the CHANGE study. We found that the prevalence of overweight and obesity in school-aged children in the rural areas of California, Kentucky, Mississippi and South Carolina ranged from 37% to 60%, with children living in Kentucky having the highest prevalence. We also found that children from these areas do not meet key obesity prevention recommendations such as screen time, sleep, and consumption of fruits, vegetables and nonfat/low-fat milk. Although we expected obese children to be more likely to engage in unhealthy behaviors, we found the opposite to be true whereby parents of children who are obese report that their child is more likely to eat ≥ 2 servings of vegetables, drink less whole milk, and been told by a doctor that they are overweight or obese. It is possible that these groups of respondent parents were highly aware of their child’s weight status and have been advised to change their health behaviors. Perhaps given the opportunity to participate in an intervention study in combination with a physician recommendation could have resulted in actual behavior change.

The prevalence of obesity in our sample was 29%, which is higher than the national average for 6–11 year old children (18-20%) [1]. The percentage of children in our sample that were at or above the 97^th^ percentile was also much higher than nationally reported data (23% vs. 14.5%) and higher than the prevalence reported for ethnic minorities. Compared to other studies completed in rural areas of America, the percentage of overweight and obese children in our sample is similar ranging from 47-54% of overweight and obesity in the different states [3] although studies have not further classified children at or above the 97^th^ percentile. Mimicking national trends, but clearly exceeding them, this rural sample has a high percentage of overweight and obese children, particularly for children above the 97^th^ percentile.

Our finding that most children regardless of weight status are not meeting health recommendations associated with obesity prevention is consistent with prior research. One study with a nationally representative sample of 2,964 children ages 4–12 years found that 65.0% (95% CI, 61.4% to 68.5%) had higher than recommended screen-time exposure, defined as ≥2 hours of combined screen-time per day [22]. Our results are the same with 65% of parents reporting that their child views more than 2 hours of screen-time per day. This represents a large proportion of children who are engaging in sedentary activities and being exposed to advertisements of high-calorie, low-nutrient-density food and beverage products. We also found that almost three quarters of the children’s parents reported that their child sleeps less than the recommended 10 hours per night. The average number of hours of sleep reported by the parents in this sample was 9.4; a poll directed by the National Sleep Foundation among 1,437 adults showed the same results in that they reported that their children slept an average of 9.4 hours per night [23].

Approximately 40% of parents reported that their child eats less than 2 servings of fruits and vegetables per day compared to the average 2 servings of fruits and 2.5 servings of vegetables that are recommended [24]. This is also similar to what others have found in that most children do not consume the recommended daily servings of fruits and vegetables to support a healthy diet. A 2010 study of 5–11 year old children (n=3761) enrolled in the Third National Health and Nutrition Examination Survey found that on average, children consume 1.4 servings of fruits and 2.4 servings of vegetables per day [25]. Another recent study reported that households below 350% of the federal poverty level were at higher risk for consuming energy-dense fruits and vegetables (i.e. fruit juice and french fries) [24]. Overall, our findings suggest that most children do not meet recommendations for physical activity, screen time and diet. Our study shows that rural children over consume calories from soda and sugar sweetened beverages, which is similar to findings from other studies. Almost all of the parents reported that their child consumes at least one soda and one sugar-sweetened beverage per day. Given that each additional 8-oz serving of sugar-sweetened beverage can correspond to an increase of 106 calories per day [26], rural children in the CHANGE Study may be consuming up to 200 discretionary calories per day from beverages. If this is over and above caloric requirement for normal growth and development, it could help explain the high rates of overweight and obesity seen in the population.

Finally, we found that obese children compared to those who are within a healthy weight range are more likely to report engaging in some healthier behaviors such as eating more servings of vegetables per day, and drinking less whole milk. Additionally, these children were more likely to have been told by a physician that they were overweight or obese. It is clear that differences across states and racial/ethnic groups exist, however we still found significant results adjusting for these important variables. Although our findings were unexpected and appear to be operating in the opposite direction, it may be that this possibly biased group of parents and children were already aware and motivated to improve health behaviors at the commencement of the study (the 36% which responded). Perhaps that by just enrolling in an intervention study in combination with receiving recommendations from their physicians; these parents of obese children had already started to improve their health behaviors. According to the transtheoretical model of behavioral change [20], this group of parents may have been at the contemplation or preparation stage and enrolling in a study helped them move to an action stage to engage in new behaviors. These motivated parents of overweight and obese children are most likely being influenced by the increased awareness of obesity in their surroundings. A qualitative study of ninth graders in rural Appalachia found that the students were quite familiar with the problem of childhood obesity. Their awareness about the rising rates of type 2 diabetes and cardiovascular disease, accompanied by personal experiences of affected family members in this region, created a fear about becoming obese [12]. Another qualitative study completed with fourth grade students, teachers and parents in rural Appalachia found similar results in that there was a heightened concern for childhood obesity and they supported the idea of their schools doing more to improve diet and physical activity. We did find, however, that parents of obese children were less likely to discuss fruit and vegetable consumption a lot/sometimes vs. never. Conceivably by being in an action stage phase of behavior change, they engaged in less conversation around fruits and vegetables as their children were already beginning to change their behaviors and did not need this extra reminder. It is also possible that parents of obese children are misreporting; literature suggests that weight status influences the accuracy of dietary reports made by children and their parents [19,27]. Parents of these obese children may be over reporting healthy behaviors and underreporting unhealthy behaviors.

Although most of the literature has shown that obese children are more likely to engage in unhealthy behaviors, we believe these preliminary findings may be an indication of an increased awareness around childhood obesity among certain families and their doctors in this region. In addition, nationally representative data from 2009–2010 show that obesity rates have stabilized [28]; it is possible that many of the community wide interventions and awareness campaigns to reduce overweight and obesity are having an effect in these more obese families. This shows promise for future interventions to have a greater impact on improving health behaviors when individuals are targeted at the right stage of change. If individual level changes are indeed occurring, it points towards the need to make improvements within the greater environment (access to healthy food and recreation centers) in order to successfully curb the obesity epidemic in rural areas.

Some limitations of this study are worth noting. First, this is a cross-sectional analysis therefore assumptions about causality cannot be made. Second, parents in this study were proxy reporters of some of their children’s health behaviors; hence they are prone to error, particularly since they are not with their children during school hours. However, studies provide support for the use of parent responses in observational studies of children [29-33]. Lastly, the response rate to our parent survey was low and those parents who responded to the survey significantly differed in the following ways: those who lived in Kentucky were less likely to respond compared to those living in California (OR=0.5, 95% CI 0.3-0.6) and those that lived in Mississippi were more likely to respond compared to those living in California (OR=1.5, 95% CI 1.1-2.0); and parents who had daughters were less likely to respond (OR=0.8, 95% CI 0.6-0.96). Within this group we still see a high percentage of overweight and obese children with varying degrees of socioeconomic backgrounds and race/ethnicities.

## Conclusions

We conclude that child overweight and obesity rates are disproportionately high in this diverse rural sample where half of rural children are overweight or obese compared to national rates of nearly one third. Most children are not meeting dietary, physical activity and screen-time recommendations. The parents of the overweight and obese children, who responded to the study survey, appear to be aware of this problem, which may be conveyed by their health care providers. Although future research is needed to confirm these findings, it is possible that participating in a research study may have initiated some of the healthier lifestyle behaviors that they reported, compared to their healthy-weight peers. If individual level changes are to be sustained in these low-income rural areas, it is crucial to also craft environmental modifications to support a healthy weight. These results also suggest that particular attention should be given to children who are within a healthy weight range to prevent obesity from developing given the prevalence of unhealthy behaviors in this sample. Future interventions and policies should continue to work toward the prevention and reduction of obesity through a community based model whereby communities and individuals are being targeted.

## Competing interest

We certify that this manuscript is an original work which has not been published elsewhere and is not under consideration for publication elsewhere. The authors do not have any conflicts of interest, financial or otherwise. All the authors listed have read the manuscript, agree that the manuscript is ready for submission to a journal and are willing to accept responsibility for the manuscript’s contents.

## Authors’ contributions

All the authors contributed to the various stages of this study. AT contributed to the study design, performed some of the statistical analysis, and drafted the manuscript. KC contributed to the study design and helped with statistical analysis. RH and VK participated in the design of the study and revised manuscript. AH and JB collected data and revised manuscript. JK and SC participated in the design of the study and revised manuscript. CDE conceived of the initial idea of the study, contributed to design of the study, revised the manuscript and contributed especially to the intellectual content. All the authors read and commented on the drafts and approved of the final version for submission.

## Pre-publication history

The pre-publication history for this paper can be accessed here:

http://www.biomedcentral.com/1471-2431/12/102/prepub
